# Endogenous opioid dependence after intermittent use of glucose, sodium chloride, and monosodium glutamate solutions

**DOI:** 10.1002/fsn3.1120

**Published:** 2019-08-15

**Authors:** Sergey K. Sudakov, Natalia G. Bogdanova, Elena V. Alekseeva, Galina A. Nazarova

**Affiliations:** ^1^ PK Anokhin Research Institute of Normal Physiology Moscow Russia

**Keywords:** endogenous opioid dependence, naloxone, pleasant taste, rats, withdrawal signs

## Abstract

In 2002, Colantuoni et al described the possibility of dependency in rats after intermittent, excessive consumption of a 25% glucose solution over a one‐week period. We hypothesized that the intermittent consumption of any tasty solution can lead to endogenous opioid dependency. Another aim was to determine whether dependency is connected to the taste of the consumed substance or with its physiological significance. Rats were maintained on chow and cyclic glucose, NaCl, or monosodium glutamate (MSG) solution for 8 days. On day 9, after a 12‐hr deprivation period and administration of intraperitoneally (IP) naloxone, the general withdrawal index was calculated as the sum of teeth chattering, head shaking, forepaw tremors, and wet dog shakes. Motor activity was also documented. After the intermittent consumption of any tasty solution, rats were found to demonstrate signs of endogenous opioid dependence. The development of dependence was not related to taste preferences or the amount of solution consumed. Intermittent use of essential substance with a pleasant taste, as glucose and sodium chloride, leads to the rapid development of endogenous opioid dependence. Withdrawal syndrome following the consumption of MSG solution is associated with the presence of sodium ions in the MSG molecule.

## 1 INTRODUCTION

Animals have five different types of taste receptors including bitter, sour, salty, sweet, and umami (O'Mahony & Ishii, 1986). These taste receptors are located on the tongue and in the oral cavity and pharynx. Molecules bind to taste receptors when food or drink is consumed thereby transmitting information from these receptors to the cerebral cortex in the brain, facilitating the sensation of taste. Bitter and sour tastes are generally perceived as unpleasant and often represent low quality or dangerous food and liquids Food or drink with a salty, sweet, or umami taste in usually perceived as pleasant. It is well known that the consumption of substances with a pleasant taste results in positive emotional sensations. This leads to the activation of positive reinforcement mechanisms including the release of beta‐endorphin in the ventral tegmental area, removal of GABA inhibitory effects on dopamine neurons, and the release of dopamine from the terminals located in different limbic and cortical structures (Arnt & Scheel‐Krüger, 1979; Heffner, Hartman, & Seiden, 1980; Jain, Mukherjee, & Singh, 2004; Maracle, Normandeau, Dumont, & Olmstead, 2019; Xenakis & Sclafani, 1982). The stimulation of the positive reinforcement system can lead to the formation of psychological and physical dependence associated with significant changes in the endogenous opioid system in particular (Wise & Bozarth, 1985). The constant use of sucrose, sodium chloride, and other substances with a pleasant taste however does not lead to symptoms of physical dependence, likely due to adaptation of taste receptors and a corresponding decrease in positive reinforcing effects of the tasty substance (Colantuoni et al., 2002; DuBois, 2016). In daily life however, consumption of nutrients and liquids with a pleasant taste occurs periodically, which can theoretically result in dependence on such products. at al. in 2002 described the possible development of a dependency in a rat after intermittent, excessive consumption of a 25% glucose solution over a one‐week period. After depriving the rat of glucose and administering a fairly large dose of naloxone, behavioral signs were observed. These are usually observed when opiates are withdrawn in opiate‐dependent animals. Authors from the same university also (Avena, Bocarsly, Rada, Kim, & Hoebel, 2008) described the possibility of endogenous opioid physical dependence after the intermittent use of a 10% sucrose solution over a one‐month period.

In this study, we hypothesized that intermittent consumption of any tasty solution can produce endogenous opioid dependency. We aimed to determine whether the formation of dependence is connected with the taste of the consumed substance or with its physiological significance.

## 2 MATERIALS AND METHODS

The experiment was performed according to the method described by Colantuoni et al. (2002) on 120 Wistar male rats weighing approximately 200–220 grams (g). Rats were housed in individual, ventilated cages (Tecniplast) on a 12:12 hr light/dark cycle. Water and laboratory chow (“Profgryzun,” Russia, 3 kcal/g, the content of sodium ions—0.2%) were available ad libitum. The rats in the experimental groups were maintained on a daily cycle of laboratory chow and 1,390 mM (25%) aqueous glucose (*n* = 10), 30 mM (0.175%) (*n* = 20), 60 mM (0.351%) (*n* = 20), or 120 mM (0.701%) (*n* = 20) of sodium chloride; or 30 mM (0.55%) (*n* = 10), 60 mM (1.1%) (*n* = 10), or 120 mM (2.2%) (*n* = 10) MSG available for 12 hr each day beginning four hours after the onset of the dark period. They were also deprived of food for the other 12 hr each day. The control group received water and chow ad libitum.

We used the same concentration of glucose solution as Colantuoni et al. (2002). Three different concentrations of sodium chloride were chosen as rats under normal conditions prefer hypotonic to normotonic sodium chloride solutions (Greenwood, Greenwood, Paton, & Murphy, 2014; Omouessi et al., 2016). Solutions of MSG were isomolar compared to solutions of sodium chloride. In addition, rats are reported to prefer such solutions to water (Smriga & Torii, 2000). Larger concentrations of sodium glutamate in rats were not preferable in the two‐bottle test (Tordoff, Alarcon, & Lawler, 2008).

Rats were maintained on cyclic glucose, NaCl, or MSG and chow for 8 days, and control rats received ad libitum chow. On day 9, after the usual 12‐hr deprivation period, instead of receiving glucose, NaCl, MSG, and chow, the rats were placed in individual tilt cages for 20 min and observers blind to the experimental condition counted the baseline frequency of rearing, grooming, cage crossing, teeth chattering, head shaking, forepaw tremors, and wet dog shakes. The general withdrawal index was calculated as the sum of all indicated signs. Motor activity was also documented. Thirty minutes before the test, 10 rats from the control group and 10 rats from each of the other groups received NaCl solutions, administered by saline [1 ml/kg IP], and all other rats were injected by naloxone [20 mg/kg IP in 1 ml/kg of saline]. Control rats in the ad libitum chow group were also food‐deprived for 12 hr. The 12‐hr period of food deprivation in the control group was included to exclude the possibility that acute food deprivation resulted in the observed effects.

Statistical analysis was performed by two‐way ANOVA followed by post hoc Bonferroni test.

## 3 RESULTS

**Figure 1:**
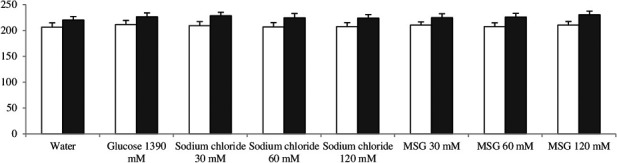
Weight gain of treated rats (grams). First day (light bars) and 8 day (dark bars)

Wistar rats were shown to prefer sucrose, sodium chloride, and MSG compared to water. Most of the rats preferred the 1390 mM glucose solution, followed by the 120 mM and 60 mM sodium chloride solutions (Table 1). The weight of the animals in the groups did not differ throughout the duration of the experiment (Figure 1).

**Table 1:** Dynamics of solution consumption of substances during day time hr (8.00–20.00) for 8 days of the experiment and the coefficient of preferences on the 8th day of the experiment

*N*		1 day	2 day	3 day	4 day	5 day	6 day	7 day	8 day	coefficient of preferences
1	Water	15.9 ± 1.8	21.4 ± 1.3	23.6 ± 1.6	22.6 ± 1.1	23.9 ± 1.3	22.4 ± 1.4	25.2 ± 1.9	25.8 ± 1.4	
2	Glucose 1,390 мM	44.0 ± 2.4*	50.3 ± 3.3*	64.4 ± 5.6*	63.2 ± 5.4*	60.4 ± 2.5*	64.5 ± 3.4*	92.0 ± 6.0*	99.5 ± 5.7*	0.9 ± 0.1
3	Sodium chloride 30 мM	22.7 ± 1.3	26.0 ± 1.7	29.8 ± 2.1	29.9 ± 2.2	32.4 ± 2.2	29.2 ± 2.5	32.2 ± 2.5	34.3 ± 1.9	0.75 ± 0.1
4	Sodium chloride 60 мM	32.4 ± 2.9*	36.0 ± 3.1*	35.2 ± 2.4*	37.6 ± 2.8*	39.7 ± 2.1*	35.2 ± 2.9*	42.4 ± 2.8*	56.6 ± 2.6*	0.8 ± 0.1
5	Sodium chloride 120 мM	44.4 ± 4.5*	48.3 ± 4.9*	47.4 ± 4.5*	44.1 ± 4.3*	50.8 ± 4.7*	53.4 ± 4.3*	55.6 ± 4.4*	51.2 ± 4.5*	0.83 ± 0.1
6	MSG 30 мM	19.6 ± 1.1	21.5 ± 1.6	24.2 ± 1.0	17.5 ± 1.6	18.9 ± 1.7	22.7 ± 2.0	24.6 ± 1.8	28.5 ± 1.4	0.61 ± 0.1
7	MSG 60 мM	20.4 ± 1.7	21.4 ± 1.4	23.9 ± 1.0	20.3 ± 1.2	19.1 ± 1.2	21.2 ± 1.6	25.4 ± 1.2	27.3 ± 1.3	0.66 ± 0.1
8	MSG 120 мM	20.9 ± 1.4	19.8 ± 1.3	17.7 ± 1.2	17.8 ± 1.6	18.9 ± 1.1	22.3 ± 1.4	23.7 ± 1.3	24.7 ± 1.5	0.59 ± 0.1

* *p* < .05 compared with water‐drinking animals.

Control animals that consumed only water after intraperitoneal administration of an isotonic solution of sodium chloride showed only teeth chattering and shaking of the head. After the administration of naloxone, rats showed teeth chattering, head shaking, and “wet dog” shaking (Table 2). However, the total “withdrawal” index in control animals, administered with naloxone and sodium chloride solution, did not differ (Table 2).

**Table 2:** The number of withdrawal signs in experimental groups

		Teeth chattering	Forepaw tremors	Head shaking	Wet dog shakes	Total index
1	Water (IP saline)	0.2 ± 0.01	0	0.6 ± 0.15	0	0.8 ± 0.2
	Water (IP naloxone)	0.3 ± 0.01	0	0.3 ± 0.15	0.2 ± 0.05	0.8 ± 0.2
2	Glucose 1,390 mM (IP naloxone)	1.4 ± 0.2*	0	4. .3 ± 0.8*	0.10 ± 0.02	5.8 ± 1.0*
3	Sodium chloride 30 mM (IP saline)	0.5 ± 0.1	1.5 ± 0.8	0	0.9 ± 0.2*	2.9 ± 1.1*
	Sodium chloride 30 mM (IP naloxone)	0.1 ± 0.01	0	2.9 ± 0.3*	1 ± 0.2*	4.0 ± 0.5*
4	Sodium chloride 60 mM (IP saline)	1.5 ± 0.4*	0.3 ± 0.2	2 ± 0.1*	0.8 ± 0.2*	4.6 ± 0.9*
	Sodium chloride 60 mM (IP naloxone)	0.7 ± 0.1	0	1.9 ± 0.4*	3.2 ± 0.4*	5.8 ± 0.9*
5	Sodium chloride 120 mM (IP saline)	1.2 ± 0.3*	0	1.6 ± 0.4*	0.2 ± 0.08	3.0 ± 0.8*
	Sodium chloride 120 mM (IP naloxone)	1 ± 0.2*	0	5.8 ± 1.3*	3.3 ± 0.5*	10.1 ± 2.0*
6	MSG 30 mM (IP naloxone)	1.3 ± 0.3*	0	3.2 ± 0.7*	0.1 ± 0.04	4.6 ± 1.0*
7	MSG 60 mM (IP naloxone)	1.8 ± 0.2*	0	3.0 ± 0.6*	0.2 ± 0.05	5.0 ± 1.0*
8	MSG 120 mM (IP naloxone)	2.5 ± 0.5*	0	2.5 ± 0.4*	0.7 ± 0.1*	5.7 ± 1.0*

* *p* < .05 compared with water‐drinking animals.

Animals that consumed glucose for 12 hr a day after the naloxone injection showed significant withdrawal signs of endogenous opioid dependence (Table 2).

**Figure 2:**
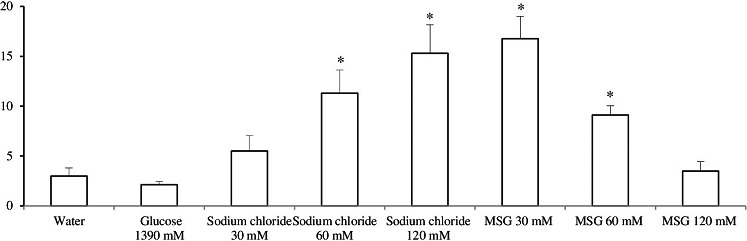
The latent period (s) of the first movement * ‐ *p* < .05 compared with the group of animals that received water

**Figure 3:**
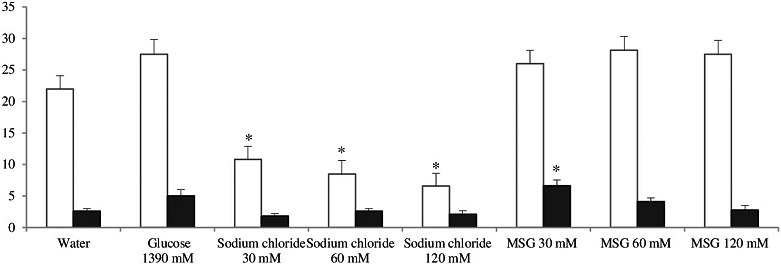
Total motor activity (crossed squares) (light bars) and exploratory activity (rearings) (dark bars) * ‐ *p* < .05 compared with the corresponding group of animals treated with water

However, the latent period of the first movement in the open field, the general motor activity, and the number of rears in animals that consumed glucose did not differ significantly from the control group (Figure 2 and 3).

Animals that consumed sodium chloride solutions also showed significant signs of withdrawal. Moreover, the higher the concentration of sodium chloride solution consumed, the higher the total withdrawal index (Table 2).

In this group, the largest number of “wet dog shakes” was observed (Table 2). The motor activity of animals that consumed sodium chloride solutions was reduced compared to the controls. The latent period of the first movement was significantly longer in the groups that consumed 0.375% and 0.71% solutions of sodium chloride (Figure 2 and 3).

After drinking sodium chloride solutions, animals demonstrated withdrawal signs even without precipitation by naloxone, but the withdrawal index was significantly lower compared to naloxone‐injected animals (Table 2). Motor activity of those animals did not differ from the controls, although the latent period of the first movement was increased (Figure 2 and 3).

Animals that consumed MSG also showed more pronounced signs of withdrawal than the rats in the control group (Table 2). The greater the concentration of MSG consumed by the animals, the bigger the withdrawal index. However, significant differences in the total index were not found between animals that consumed MSG solutions of different concentrations. Animals in these groups had the greatest number of head shakings, and teeth chattering was also observed. The latent period until the first movement in the open field in rats that consumed 30 mM solution of MSG was significantly longer than in control animals. An increasing concentration of MSG was associated with shortening of the latent period. Animals that consumed 120 mm of MSG did not differ from the controls (Figure 2). Also rats that consumed the 30 mM solution of MSG demonstrated an increased number of rears (Figure 3). Total motor activity in animals that consumed MSG did not differ from controls (Figure 3).

## 4 DISCUSSION

It is known that rats prefer sweet and salty solutions to water. Moreover, the preference depends on the concentration of such solutions. Animals prefer hypotonic and normotonic, rather than hypertonic solutions of sodium chloride (De Luca, Pereira‐Derderian, Vendramini, David, & Menani, 2010; Greenwood et al., 2014). Rats no longer prefer solutions containing more than 300 mM of MSG. The preference for sugar solutions is not so dependent on their concentration (Inui‐Yamamoto et al., 2017).

Our study showed that the preferred solution for Wistar rats was glucose. The preference ratio, which is the ratio of the solution consumed to the total amount of liquid consumed, in rats that consumed glucose solution on the eighth day was higher than that seen in animals from the other groups. We identified signs of opiate‐like withdrawal syndrome in animals that consumed glucose solution. However, similar earlier studies (Colantuoni et al., 2002) have shown a much more pronounced withdrawal syndrome with a large number of observed signs. Firstly, the differences may be related to genetic factors, as we used Wistar rats in our study rather than Sprague–Dawley rats In addition, the rats in our study were likely younger than those used in previous studies, the latter estimated on the basis of their initial weight (Colantuoni et al., 2002) (from 300 to 475 g). It is possible that young animals develop opiate dependence more slowly, and manifestations of the withdrawal syndrome are significantly less.

Administration of naloxone dramatically increased withdrawal after the consumption of sodium chloride solution; however, signs of withdrawal were also observed without naloxone precipitation. This suggests that opioid mechanisms form the basis of the observed withdrawal syndrome.

Despite having the highest preference ratio, the withdrawal index in animals that consumed glucose solution was significantly lower than in rats that consumed 120 mM sodium chloride solution. In addition, we found no significant correlations between the coefficient of preference and the severity of the withdrawal syndrome in any of the experimental groups. This suggests that the development of dependence is not related to the taste preferences and the amount of the preferred solution drunk. The severity of sodium chloride or glucose dependence likely relates to their biological role, sodium ions being a substrate for excitable tissues, and glucose being an energy substrate. It is possible the need for sodium ions is more significant. The need for glutamate however is not critical; therefore, the withdrawal syndrome after consumption of sodium glutamate solutions may be associated with the presence of sodium ions in the MSG molecule. However, the development of endogenous opioid dependence on MSH requires further study. It remains inexplicable that the highest concentration of MSH, in contrast to the isomolar concentration of sodium chloride, causes the smallest effect on the latent period of the first movement. Moreover, our unpublished data suggest that the use of MSH for 28 days according to the method described in this article may cause the formation of psychological dependence. This does not happen when rats use isomolar solutions of sodium chloride.

There are some limitations to this study. Firstly, the dose of naloxone used was too high. We chose the same dose of naloxone as was used in the Colantuoni study (2002). We did not try further doses, but certain groups of animals that were drinking sodium chloride solution demonstrated signs of endogenous opiate dependence even without naloxone precipitation. Secondly, the withdrawal syndrome observed in our study was weak and incomplete compared to the withdrawal syndrome in exogenous opiate‐dependent rats. In our study, we did not observe convulsions, diarrhea, rhinorrhea, and posture disturbances. These symptoms are often observed during withdrawal in opiate dependence.

In conclusion, the intermittent use of essential substances with a pleasant taste like glucose and sodium chloride leads to rapid development of endogenous opioid dependence. Whether endogenous opioid dependence develops when glutamate is consumed is an open question and requires further study.

## ETHICAL APPROVAL

The authors confirm the absence of any conflicts of interest. Study's protocols and procedures were ethically reviewed and approved by P.K. Anokhin Research Institute of Normal Physiology Animal Care and Use Committee (Permission number 231) and conform to Directive 2010/63/EU. Human testing is unnecessary in this study.
